# Physiological State Influences the Social Interactions of Two Honeybee Nest Mates

**DOI:** 10.1371/journal.pone.0032677

**Published:** 2012-03-09

**Authors:** Geraldine A. Wright, Joshua L. Lillvis, Helen J. Bray, Julie A. Mustard

**Affiliations:** 1 Centre for Behaviour and Evolution, Institute of Neuroscience, Newcastle University, Newcastle upon Tyne, United Kingdom; 2 Neuroscience Institute, Georgia State University, Atlanta, Georgia, United States of America; 3 School of Biology, Newcastle University, Newcastle upon Tyne, United Kingdom; 4 School of Life Sciences, Arizona State University, Tempe, Arizona, United States of America; Cajal Institute, Consejo Superior de Investigaciones Científicas, Spain

## Abstract

Physiological state profoundly influences the expression of the behaviour of individuals and can affect social interactions between animals. How physiological state influences food sharing and social behaviour in social insects is poorly understood. Here, we examined the social interactions and food sharing behaviour of honeybees with the aim of developing the honeybee as a model for understanding how an individual's state influences its social interactions. The state of individual honeybees was manipulated by either starving donor bees or feeding them sucrose or low doses of ethanol to examine how a change in hunger or inebriation state affected the social behaviours exhibited by two closely-related nestmates. Using a lab-based assay for measuring individual motor behaviour and social behaviour, we found that behaviours such as antennation, willingness to engage in trophallaxis, and mandible opening were affected by both hunger and ethanol intoxication. Inebriated bees were more likely to exhibit mandible opening, which may represent a form of aggression, than bees fed sucrose alone. However, intoxicated bees were as willing to engage in trophallaxis as the sucrose-fed bees. The effects of ethanol on social behaviors were dose-dependent, with higher doses of ethanol producing larger effects on behaviour. Hungry donor bees, on the other hand, were more likely to engage in begging for food and less likely to antennate and to display mandible opening. We also found that when nestmates received food from donors previously fed ethanol, they began to display evidence of inebriation, indicating that ethanol can be retained in the crop for several hours and that it can be transferred between honeybee nestmates during trophallaxis.

## Introduction

Homeostatic mechanisms for the maintenance of physiological state regulate many important behavioural processes in animals including foraging for and consuming food. Solitary animals search for food in a cycle that mirrors their nutritional state: hungry animals are more likely to forage and to move, and satiated ones are more likely to be quiescent after eating [1]–[4]. In contrast, in social insect societies such as bee or ant colonies, food seeking behaviour is largely carried out by specialist foragers who continually forage such that they collect food and return to share it with other colony members. Although adult bees feed directly from colony stores of pollen or honey, foraging worker honeybees use a portion of the food they collect or they are often given food from nestmates (e.g. nurses) rather than eating honey reserves (reviewed in Crailsheim, 1998) [5]. Within colonies, food sharing between adults is facilitated by behavioural cues such as begging [6]; as a result of food solicitation, trophallaxis, or food sharing, occurs between the begging ant or bee and the donor from whom it solicits food. When entire colonies are starved, begging between nestmates increases [7]–[9]. Food sharing is also an important way that nestmates communicate their own hunger state to other nestmates to aid in the regulation of foraging within the colony [7], [10]. Whether or not hunger state influences the dynamic of food sharing beyond an increase in food solicitation is not well-understood.

Invertebrate animals are often studied to understand how neural circuits give rise to behaviour. One of the main contributions of invertebrates has been in providing insight into the molecular mechanisms underlying the effects of human-abused drugs on the brain. In *Drosophila, C. elegans* and honeybees, motor function, learning and memory, communication, and reward seeking are all affected by exposure ethanol [11]–[18]. As models, they have been successful because the molecular targets of ethanol in neural circuits in mammals are also expressed in the insect nervous system [19].

While it is clear that intoxication with drugs such as ethanol leads to changes in individual behaviour, we know less about how the change in state that accompanies drug consumption or exposure influences social interactions among animals. For example, when animals consume ethanol, inebriation could lead to fewer social interactions and social isolation if inebriated individuals fail to communicate or interact appropriately. Intoxication with drugs or toxic substances could also affect food sharing behaviour in social insects if intoxicated individuals are unwilling to share food with others or unmotivated to eat as a result of consuming ethanol. Only a few studies to date have examined social interactions in intoxicated or inebriated insects [14], [20]–[22]. The experiments carried out to understand how ethanol influences social interactions in honeybees have reported conflicting results [21], [22].

Here, we investigated how hunger and inebriation state influence food sharing behaviour between two honeybee nestmates. Using a newly developed assay for measuring individual and social behaviours in pairs of honeybees, we aimed to identify whether the physiological state of food donors produced predictable patterns of reaction to begging behaviour by hungry nestmates. What distinguishes our experiments from all preceding studies of social interactions between honeybee nestmates is that we controlled the satiety state of all bees by restraining them and feeding them 1.0 M sucrose 24 h prior to the assay. At the start of the assay, the donors were fed defined amounts of sucrose solution to standardize each donor's satiety state. By doing this, we controlled variation in satiety state that allowed us to observe clear differences in the behaviour of hungry and fed bees. We expect that the hungry bees in our study were in a greater state of food deprivation, and that this control over state combined with the detailed observation of interactions between two individuals made it possible to distinguish the state-induced changes in all behaviours we recorded. Our study emphasizes the importance of carefully defining physiological state prior to measuring nestmate interactions. In a previous study of the influence of ethanol on honeybee motor function, we characterized inebriation using a suite of behaviours including grooming behaviour and the righting reflex. We measured motor function as before but also included behaviours that described social interactions between the two bees assayed. To identify whether inebriation state influenced the extent to which donors were willing to share food, we also measured the amount of food transferred to the hungry nestmate and observed receivers in order to identify whether ethanol received via trophallaxis had an effect on their behaviour.

## Methods

### Subjects

The study was performed using honey bees (*Apis mellifera*) collected from indoor colonies during Oct-Dec 2004 and from outdoor colonies during June 2005 at the Rothenbuhler Honeybee Laboratory at Ohio State University. Returning foraging workers were captured from the colony entrance using glass vials. In the lab, they were subjected to cooling anaesthesia and restrained using standardised techniques [23]. Once harnessed, each subject was fed 18 µl of 2.0 M sucrose, and kept at room temperature for 18–24 h prior to being used in an experiment.

### Behavioural Observations

**Figure 1:**
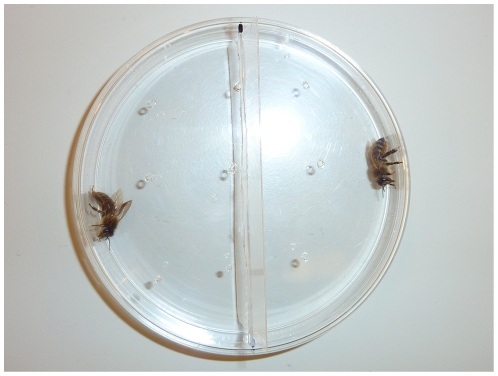
The observation arena. The bees were placed in the petri-dish separated by a plastic partition for 10 min prior to the experiment.

Two hours prior to the observation, each bee was fed 9 µl of one of the following treatments: 1.0 M sucrose, 5% ethanol in 1.0 M sucrose, or 10% ethanol in 1.0 M sucrose. (The sucrose concentration was held constant across all treatments.) Just prior to the observation, bees that were designated as the ‘donor’ were given 20 µl 1.0 M sucrose; the ‘receiver’ bees were not fed again. In the ‘no food’ treatment, the donor was not given the 20 µl 1.0 M sucrose prior to the observation. Observational arenas were composed of 100×15 mm plastic Petri dishes (Figure 1). The lid of the petri dish was cut in half to allow for a cut-to-fit piece of plastic to be placed between the two halves of the lid to separate the two bees. Both bees were briefly cooled and the bee that would be the focus for the observation was marked on the thorax with white out. The bees were then weighed and placed either side of the divider; they remained separated for 10 min to allow them acclimate to their surroundings before the divider was removed and the 30 min observation period began. All observations occurred between 2–7pm by JLL using The Observer software (Noldus Information Technology). We classified behaviour as either ‘individual’ behaviour (as described in Maze *et al.*, 2006) [15] or social behaviour. Individual behaviours included the following: walking, grooming, stopped and upside down (fanning and flying behaviour, as reported in Maze *et al.* 2006, were not reported or analysed here because they were very infrequently observed). Social behaviours included: antennation, proboscis extension/licking (begging), trophallaxis, and mandible opening. After the observation period, both bees were removed from the observation arena and reweighed to confirm that fluid had been transferred during trophallaxis. (Whether or not the bee was giving or receiving trophallaxis could not be easily determined, so these two behaviours were grouped into a single behavioural category).

To determine whether the receiver bees obtained ethanol from the donors during trophallaxis, we also observed the behaviour of receiver bees when the donor had been given 20 µl of either 1.0 M sucrose or 1.0 M sucrose containing 5% ethanol prior to the observation. These observations were carried out as described above.

### Statistical Analysis

All statistics were calculated using the software, SPSS. The observational data were compiled for 5 min time intervals and for lag sequential analysis using The Observer software and are reported in the file Data S1. We analysed the ontogeny of the behaviours in our 30 min interval for each behaviour using a repeated-measures general linear model (GLM) for the standardized amount of time engaged in the behaviour for each interval (as a percentage) and the mean bout duration as described in Maze *et al.* (2006) [15]. Bout frequency was analysed using repeated-measures Poisson Regression (PR) [24]. The behaviour of the receivers was analysed using multivariate ANOVA (MGLM). Multiple comparisons were calculated as least square differences (lsd) and were only reported as significant if they were less than P<0.05 after a Bonferroni correction. To identify whether it was possible to predict treatment using the behavioural data for the donor bees, we first reduced the dimensionality of the observation data using a principle components method for factor analysis [25]. The resulting scores for factors with eigenvalues greater than 1 were entered into a canonical discriminant analysis and used to classify the 4 donor treatments (sucrose, 5% ethanol, 10% ethanol, no food) in our experiment [25]. Differences in the weight of donors and receivers were calculated using ANOVA.

## Results

### State influences the expression of individual motor behaviour in donor bees

**Figure 2:**
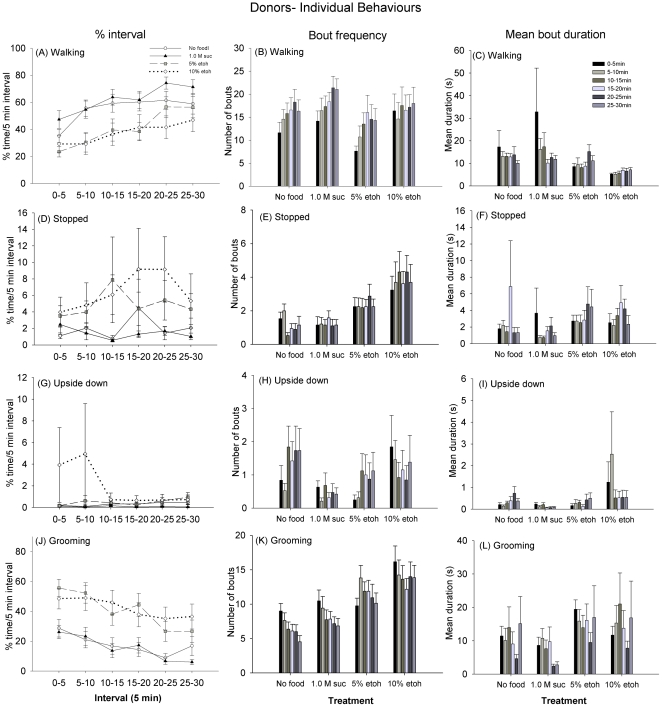
Hunger and inebriation state influence motor behaviour in donor honeybees. The time spent engaged in each of the behaviours, the mean bout frequency, and the mean bout duration are shown for each 5 min interval. (A–C) Bees spend less time walking if they had been pre-fed with a 1.0 M sucrose solution containing ethanol; the ethanol treatments did not differ (*P* = 0.381), nor did the hungry and fed bees (*P* = 0.979). Bout duration and frequency were unaffected by treatment. (D–F) The amount of time spent stopped was greater for the donors pre-fed ethanol. The frequency of bouts of stopped behaviour was the same for the hungry and sucrose-fed bees (*P* = 0.645), but the ethanol-fed bees had more frequent bouts of stopped behaviour than the controls (*P*<0.05 for all comparisons). Mean bout duration was not affected by treatment. (G–I) Ethanol treatment did not influence the total amount of time spent upside down (e.g. the ability to perform the righting reflex) but 10% ethanol-fed bees had longer and more frequent bouts of upside down behaviour (*P*<0.001 for all comparisons). Hungry bees were also more likely to perform bouts of upside down behaviour than the sucrose or 5% ethanol fed bees. (J–L) Donor bees fed ethanol spent more time grooming, and performed more bouts of grooming (*P*<0.05 for all comparisons) that lasted longer than hungry bees or those fed sucrose. N_Hungry_ = 20, N_1.0 M Suc_ = 19, N_5% etoh_ = 17, and N_10% etoh_ = 13. ± SEM Note: Y-axis scale is not the same on all graphs.

A previous series of experiments in bees established that ethanol inebriation was characterized by a suite of changes in motor function that depended on the dose of ethanol bees had consumed [15]. Using this method, we measured the same variables for the donor and receiver bees in this assay (walking, stopped, grooming, upside down). For donors, we observed that in general the time spent walking increased at later intervals (Fig. 2A, interval main effect, GLM, F_1,63_ = 13.8, *P*<0.001). However, on average, donor bees spent less time walking if they had been given an ethanol dose (main effect, GLM, F_3,63_ = 3.51, *P* = 0.020). The mean number of bouts of walking increased over time (Fig. 2B, main effect, Pois. Reg. χ_5_^2^ = 21.5, *P* = 0.001), but walking bout duration did not change over the observation period (Fig. 2C, main effect, GLM, F_1,58_ = 0.189, *P* = 0.665). Walking bout duration was on average longer for bees fed sucrose than for the other treatments (main effect GLM, F_3,58_ = 5.58, *P* = 0.002), but walking bout frequency was the same for all treatments (main effect, Pois. Reg. χ_3_^2^ = 4.47, *P* = 0.215). In contrast, the amount of time spent stopped did not vary across the intervals (Fig. 2D, main effect, GLM, F_1,63_ = 0.02, *P* = 0.883), but it was affected by treatment (main effect, GLM, F_3,63_ = 5.47, *P* = 0.002). Stopped bout frequency clearly depended on whether or not the donors had been fed ethanol prior to the assay (Fig. 2E, main effect, Pois. Reg. χ_3_^2^ = 33.9, *P*<0.001), and donors fed 10% ethanol exhibited longer bout durations (Fig. 2F, main effect, GLM, F_3,58_ = 3.54, *P* = 0.020).

Whether or not bees could right themselves (expressed as time spent upside down) and the time spent grooming were characteristic behaviours observed in inebriated bees [15]; bees fed doses of 25% or greater spend more time upside down, and bees fed 5–10% ethanol spend the most time grooming. In this study, we did not observe that donor bees fed ethanol spent more time upside down over the observation period (Fig. 2G, main effect GLM, F_3,63_ = 1.53, *P* = 0.216). However, the 10% ethanol-treated donors and the hungry donors had more frequent bouts of upside down behaviour those fed sucrose or 5% ethanol (Fig. 2H, main effect, Pois. Reg. χ_3_^2^ = 49.9, *P*<0.001). The 10% ethanol treated donors also had the longest bouts of upside down behaviour (Fig. 2I, main effect GLM, F_3,63_ = 4.41, *P* = 0.005). All of the bees spent more time grooming during the first 10 min of the observation, but the ethanol-treated donors were also more likely to spend time grooming during the entire assay (Fig. 2J, main effect, GLM, F_3,63_ = 8.99, *P*<0.001). Ethanol-treated donors also exhibited more bouts of grooming (Fig. 2K, Pois. Reg. χ_3_^2^ = 33.8, *P*<0.001) that persisted for longer during each bout than either the hungry or sucrose-fed bees (Fig. 2 L, main effect GLM, F_3,58_ = 312, *P*<0.001).

### Hunger and inebriation affect social behaviour in donor bees

**Figure 3:**
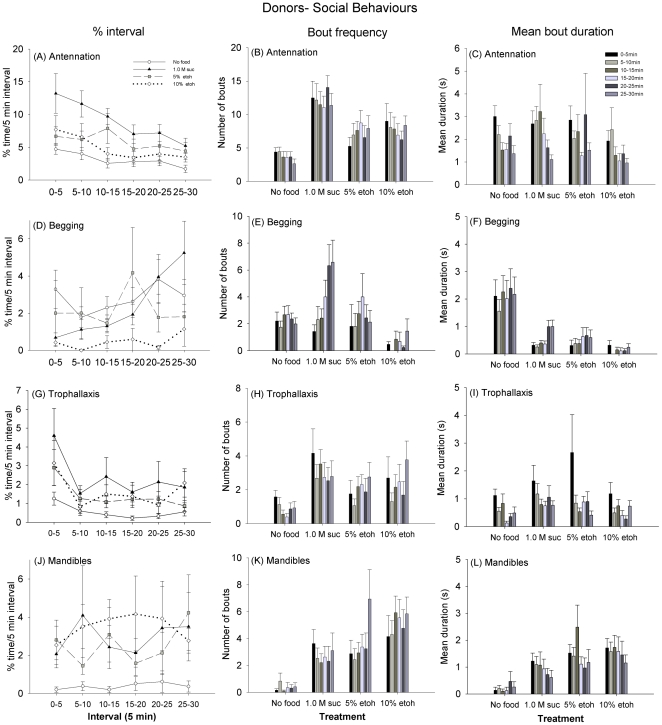
Hunger and inebriation state influence social behaviour. The time spent engaged in the each behaviour, the mean bout frequency, and the mean bout duration are shown for each 5 min interval. (A–C) Antennation was greatest at the start of the observation, and gradually decreased. The donors fed 1.0 M sucrose spent more time antennating and had longer bouts of antennation than bees in any of the other treatment groups; the hungry bees spent the least time antennating, and had the shortest bouts of antennation (*P*<0.001 for all comparisons). (D–F) Begging behaviour (proboscis out) generally increased at later intervals for all treatments; the 10% ethanol-fed bees spent the least time begging. The hungry donors exhibited the longest bouts of begging. (G–I) Hungry bees were the least likely to spend time performing trophallaxis. They also had the least frequent and shortest bouts of trophallaxis. The ethanol treatments were not less likely than the sucrose fed bees to perform bouts of trophallaxis, nor were their bouts shorter (all comparisons, *P*>0.050). (J–L) The hungry bees were also least likely to exhibit mandible opening, but the other treatments did not differ in time spent performing mandible opening (all comparisons, *P*>0.050). Bout frequency and bout duration of mandible opening was greatest for the ethanol-fed donors (P<0.010). N_Hungry_ = 19, N_1.0 M Suc_ = 19, N_5% etoh_ = 16, and N_10% etoh_ = 13. ± SEM Note: Y-axis scale is not the same on all graphs.

We characterized social interactions in these experiments as antennation, begging (proboscis out to receive food), trophallaxis, and mandible opening. Antennation is the performance of contact chemoreception and, therefore, object recognition. The donor bees fed sucrose prior to the assay (the 1.0 M sucrose treatment and both ethanol treatments) generally spent more time antennating (Fig. 3A, main effect GLM, F_3,63_ = 7.55, *P*<0.001) with more frequent bouts of this activity (Fig. 3B, main effect, Pois. Reg. χ_3_^2^ = 40.3, *P*<0.001). The sucrose-fed donors were more likely to spend time antennating than either the hungry bees (lsc, *P*<0.001) or either of the ethanol-fed bees (lsc, 5%: *P* = 0.015; 10%: *P* = 0.003). Time spent antennating was also greater during the first 15 min of the observation when the bees were first allowed to interact for all treatments (main effect, GLM, F_1,63_ = 20.0, *P*<0.001). Mean bout duration was the same for all treatments (Fig. 3C, main effect, GLM, F_1,63_ = 1.68, *P* = 0.171).

Begging and trophallaxis behaviour are the main ways that food is solicited and transferred among social insect nestmates. The amount of time spent begging depended both on whether bees were hungry or inebriated (Fig. 3D, main effect, GLM, F_3,63_ = 4.11, *P* = 0.010); the 10% ethanol-fed bees spent the least amount of time begging (*P*<0.05 comparisons with hungry and sucrose-fed bees). Donors in all of the other treatment groups begged more frequently than the 10% ethanol-fed bees (main effect, Pois. Reg. χ_3_^2^ = 10.5, *P* = 0.014). The hungry bees had the longest bouts of begging (Fig. 3F, main effect, GLM, F_3,58_ = 11.4, *P*<0.001). In fact, their bouts of begging were 2–3 times longer than any of the bees in the other treatments. All groups began to spend more time soliciting food during the last 10 min of the observation period (main effect, GLM, F_1,63_ = 11.1, *P* = 0.001).This increase in begging behaviour was most dramatic in the sucrose-fed donors, as the frequency of begging bouts increased significantly at later intervals (Fig. 2E) (interaction, Pois. Reg. χ_14_^2^ = 102, *P*<0.001).

Time spent performing trophallaxis, or food donation, was greatest at the start of the assay for all the donor bees (Fig. 3G, main effect, GLM, F_1,63_ = 5.32, *P* = 0.024); the bout duration was also longest at this time (Fig. 3 I, main effect, GLM, F_1,58_ = 11.2, *P* = 0.001). Trophallaxis bout frequency did not vary over the observation, (Fig. 3H, main effect, Pois. Reg. χ_5_^2^ = 9.20, *P* = 0.102), but the sucrose and ethanol-fed donor bees were more likely than the hungry bees to perform bouts of trophallaxis (time spent, main effect GLM, F_3,63_ = 3.53, *P* = 0.020, bout frequency, main effect, Pois. Reg. χ_3_^2^ = 15.7, *P* = 0.001). Inebriation state did not affect the time spent performing trophallaxis (lsc, *P*>0.050), and inebriated bees did not perform fewer bouts on average than the sucrose-fed bees (lsc, *P*>0.050).

Mandible opening is a form of aggression between social insect nestmates elicited to demand food [26]; in honeybees, it has also been shown to precede food donation [6]. The time spent displaying this behaviour was more or less constant over the entire observation period for all groups (Fig. 3J, interaction, GLM F_3,63_ = 0.118, *P* = 0.949), but the hungry donors were the least likely to display this behaviour of all of the treatment groups (lsc, P<0.050). The bout frequency of mandible opening increased for the inebriated bees at later intervals, but not for the hungry or sucrose-fed donors (Fig. 3K, 2-way interaction, Pois. Reg χ_15_^2^ = 54.2, *P*<0.001). The ethanol-fed donors were also most likely to exhibit bouts of mandible opening (lsd, *P*<0.05 for all comparisons). Bout duration of mandible opening was approximately as long on average as bouts of begging or trophallaxis for all bees (i.e. 2–4 s, Fig. 3L). Bouts of mandible opening were longest in duration at the beginning of the assay, when the receiver bees were also most likely to be solicit food from the donor bee (main effect GLM, F_1,58_ = 6.45, *P* = 0.014). The sucrose and ethanol-fed bees exhibited longer bouts of mandible opening than the hungry bees (main effect GLM, F_3,58_ = 10.2, *P*<0.001).

**Figure 4:**
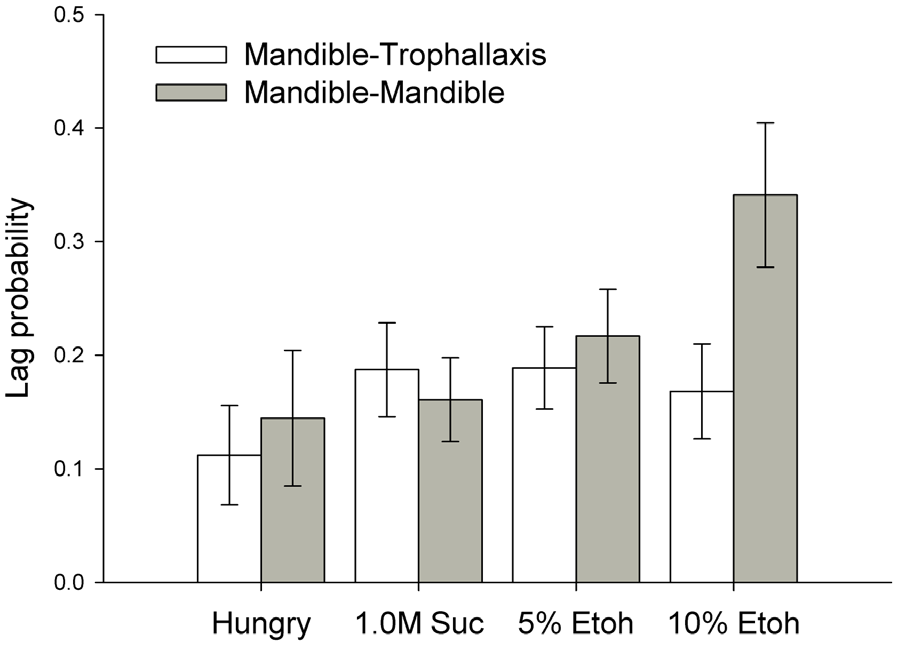
Lag sequential analysis of mandible opening behaviour of the donor bees revealed that the donors fed 10% ethanol were more likely to have bouts of mandible opening that were immediately followed by a second bout of mandible opening than bees receiving any of the other treatments. The probability that a mandible opening bout was followed by another mandible opening bout was the same for all treatments except the 10% ethanol-fed donors. N_Hungry_ = 24, N_1.0 M Suc_ = 46, N_5% etoh_ = 46, and N_10% etoh_ = 27 (N refers to number of observations of mandible opening behaviour that were followed by antennation, begging, trophallaxis, or mandible opening in each group). ± SEM.

To identify whether mandible opening behaviour was a form of food offering behaviour, we performed a lag sequential analysis to look at the pattern of behaviour immediately following mandible opening. We specifically compared whether mandible opening was followed by trophallaxis or by a second bout of mandible opening behaviour. Using lag sequential analysis with a lag of 1 (i.e. the behaviour immediately following the focal behaviour) on the bouts of mandible opening, we found that hunger and inebriation did not influence the probability of performing mandible opening followed by trophallaxis (food offering behaviour) (Fig. 4, 1-way ANOVA, F_3,142_ = 0.597, *P* = 0.618). On the other hand, inebriated bees, and in particular the 10% ethanol-fed bees, were more likely to repeatedly open and close their mandibles without performing trophallaxis (1-way ANOVA, F_3,142_ = 2.82, *P* = 0.041). (We also examined the probability that mandible opening was followed by the other social behaviours, antennation and begging. Treatment did not influence the probability of observing this sequence of behaviour for either variable, antennating: 1-way ANOVA, F_3,142_ = 1.54, *P* = 0.206; begging: 1-way ANOVA, F_3,142_ = 1.88, *P* = 0.136, data not shown).

### Social behaviour predicts hunger and inebriation state in donor bees

We tested whether it was possible to classify the satiety and inebriation state of the donor honeybees using the entire suite of behavioural variables that we measured. This analysis was performed using standardized amount of time spent per 5 min interval (mean percentage of time spent per interval). To do this, we first reduced the dimensionality of the data by performing a factor analysis (FA) on all the behavioural variables for each interval (6 intervals for 8 variables; Table S1). The factor scores generated for the 13 factors with eigenvalues over 1 were then entered into a canonical discriminant analysis (CDA).

The CDA produced two significant classification functions. The first function accounted for 50% of the variation in the data and separated donor bees fed sucrose solution containing ethanol from those fed nothing or 1.0 M sucrose (CDA, χ_39_^2^ = 77.5, *P*<0.001; Table 1). As indicated by the standardized discriminant function coefficients, this function was mainly derived from information from the first and second factors of the FA. These factors predominantly represented variation in the individual motor behaviours; for example, in F1, walking and grooming had strong factor loadings that were inversely correlated (as represented by the sign of the factor loading) and in F2, upside down behaviour was strongly represented, as indicated by the magnitude of the factor loading (Table S1). The 10% ethanol-fed bees, defined by the fact that they were less likely to walk, more likely to groom, and more likely to be upside down, were significantly separated from the bees that were not fed ethanol (No food and 1.0 M sucrose groups).

**Table 1:** Canonical Discriminant Analysis for All Behaviours.

	Discriminant function		
	1	2	3
Eigenvalue	0.888	0.628	0.253
% variance	50.200	35.500	14.300
Wilks' Lambda	0.260	0.490	0.798
Standardized CDA Function Coefficients			
**Factors**			
F1	**0.814**	0.013	0.047
F2	**−0.550**	0.403	0.290
F3	−0.153	**0.756**	−0.073
F4	0.370	**0.638**	−0.164
F5	0.055	0.246	0.042
F6	−0.118	0.115	0.282
F7	0.060	0.194	0.298
F8	−0.193	0.153	0.000
F9	−0.013	0.119	**0.549**
F10	0.478	0.021	**0.563**
F11	0.182	−0.003	−0.005
F12	0.114	−0.051	0.362
F13	0.353	0.310	−0.338
Non-standardized Function Coefficients			
**Treatment**			
No food	−0.803	−0.958	−0.074
1.0 M sucrose	−0.826	1.117	−0.056
5% Ethanol	0.825	−0.009	0.711
10% Ethanol	1.237	0.026	−0.743

The standardized coefficients in bold indicate the factors from the factor analysis that contributed the most to classification of the treatments by each of the functions (all factors contributed, but the criterion for emphasis was a coefficient greater than ±0.5). The sign and magnitude of the non-standardized coefficients indicate how the functions classified bees according to treatment. The order of the functions indicates the distance in similarity between the treatments; the line indicates the most likely split between groups made by the classification function.

The second function accounted for 35.5% of the variation and significantly separated the donor bees fed 1.0 M sucrose from the hungry, unfed bees (CDA, χ_24_^2^ = 40.9, *P* = 0.017; Table 1). This function was largely represented by data from factors 3 and 4 in the PCA (Table S1); F3 was characterized by the fact that bees that exhibited mandible opening were also less likely to beg for food (factor 3) whereas F4 predominantly represented a correlation between trophallaxis and upside down behaviour (factor 4). Notably, in this function the ethanol-fed bees had similar unstandardized function coefficients, indicating that they had very similar behaviours.

To verify our conclusions above, we performed the FA-CDA analysis on the individual (walking, stopped, upside down, grooming) and social (antennation, begging, trophallaxis, mandibles open) behaviours separately. The analysis of the individual behaviours yielded one significant classification function that, as before, separated the ethanol-fed bees from those that did not receive ethanol (CDA, χ_18_^2^ = 38.4, *P* = 0.003; Table S2). Likewise, when the FA-CDA analysis was repeated on the social variables alone, we obtained one classification function that separated the hungry bees from those fed with 1.0 M sucrose prior to the experiment (CDA, χ_23_^2^ = 43.1, *P* = 0.003; Table S3).

### Receivers display evidence of ethanol-induced changes in behaviour

**Figure 5:**
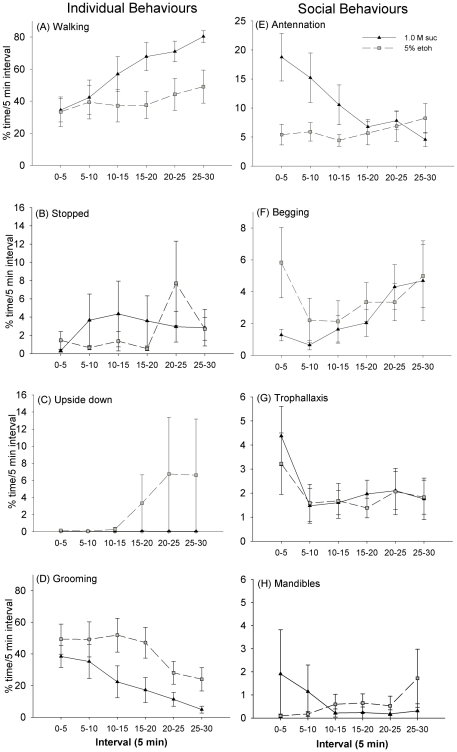
Receiver behaviour towards 1.0 M sucrose-fed and 5% ethanol-fed donors. (A–D) The receivers paired with 5% ethanol-fed donors walked less and groomed more often than the sucrose-fed donors (E–G). The only social behaviour that was affected by pairing with an ethanol-fed donor was antennation: in this case, the receiver spent less time antennating the donor over the interval than the receivers paired with 1.0 M sucrose fed donors. N_1.0 M Suc_ = 10, N_5% etoh_ = 13. ± SEM Note: Y-axis scale is not the same on all graphs.

In addition to observing state-based changes in the behaviour of donor bees, we also examined how the behaviour of receivers was affected when they interacted with either sucrose-fed or 5% ethanol-fed donors. In these observations, we specifically aimed to identify whether interacting with an ethanol-fed donor led to inebriation in the receiver. The receivers in both groups spent the same amount of time engaging in trophallaxis early in the observation (Fig. 5G, main effect, MGLM, F_1,126_ = 0.260, *P* = 0.611), indicating that food was probably transferred from the donor bee to the receiver. As expected if ethanol had been transferred to the receiver bees, the receivers that had been paired with a 5% ethanol-fed donor spent less time walking (Fig. 5A, main effect, main effect, MGLM, F_1,126_ = 11.7, *P*<0.001) and more time grooming (Fig. 5D,main effect, MGLM, F_1,126_ = 15.1, *P*<0.001) than those paired with a donor fed 1.0 M sucrose. Consistent with the behaviour of the donor bees, the amount of time spent upside down was not significantly greater in the receivers paired with the 5% ethanol donors (Fig. 5C main effect, main effect, MGLM, F_1,126_ = 2.18, *P* = 0.142), nor was the amount of time spent stopped (Fig. 5B, main effect, MGLM, F_1,126_ = 0.180, *P* = 0.672). The only social behaviour affected by whether or not a receiver was paired with a 5% ethanol donor was the amount of time spent antennating (Fig. 5E, interval×treatment, MGLM, F_5,126_ = 3.31, *P* = 0.008); time spent performing the other behaviours did not depend on the treatment (Fig. 5F,G,H, begging: F_5,126_ = 1.89, *P* = 0.171; trophallaxis: F_5,126_ = 0.260, *P* = 0.611; mandibles open: F_5,126_ = 0.009, *P* = 0.927). In general, the mean bout duration and the frequency of both individual and social behaviours were reflected in the trends seen in the percent of the time spent during the interval (Figure S1 and S2).

### Food transfer takes place during the observation period

**Figure 6:**
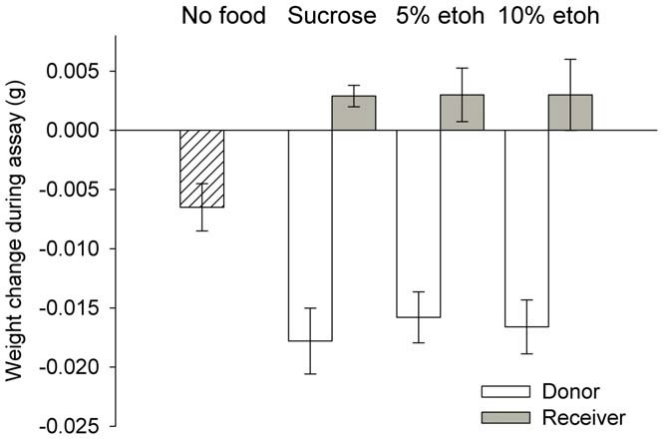
Ethanol-fed donors transferred the same amount of food on average as the sucrose-fed donors. Hungry honeybees, that had been starved 24 h prior to the assay, still transferred food, but significantly less food than those fed with 20 µl of 1.0 M sucrose prior to the assay (the 1.0 M sucrose, and 5% and 10% ethanol fed donors). N_Hungry_ = 34, N_1.0 M Suc Donors_ = 26, N_1.0 M Suc Receivers_ = 26, N_5% etoh Donors_ = 26, N_5% etoh Receivers_ = 26 and N_10% etoh Donors_ = 13, N_10% etoh Donors_ = 13. ± SEM.

To verify that the receivers actually received food during trophallactic interactions with the donors in all the experiments, we weighed both bees before and after the behavioural assay. All donors lost weight over the interval of the assay, and all receivers gained weight (Fig. 6, bee type main effect, GLM, F_1,123_ = 100, *P*<0.001). The amount of weight lost by the donors depended on whether or not they had received food prior to the assay; the ‘hungry’ donors that had not been fed, lost weight (6.5 mg), but lost significantly less weight than donors that received food (1-way ANOVA, F_3,97_ = 5.78, *P* = 0.001). Whether or not a donor had been fed ethanol prior to the assay, however, did not influence the amount of weight lost by donors or the amount of weight gained by receivers (treatment main effect, GLM, F_2,123_ = 0.41, *P* = 0.869). We, therefore, conclude that treatment with ethanol did not affect the amount of food transferred from the donor to the receiver during trophallaxis. The amount of weight gained on average by the receivers (3 mg) was not equal to that lost by the donors (16 mg).

## Discussion

Our results clearly show that hunger and inebriation state influence social interactions between two honeybee nestmates. Compared to bees provided with sucrose without ethanol exposure, hungry bees were more likely to beg for food and less likely to communicate via antennation or share food. Inebriated bees were less likely to communicate via antennation or beg and more likely to repeatedly open their mandibles, potentially as a warning display to reject begging receivers. Our data also show that substances like ethanol that are retained in the crop can be transferred between honeybee nestmates via trophallaxis.

### Hunger state and social interactions in bees

Food is an important currency in social insect colonies, and the effect of hunger on social interactions has been studied previously to identify how the quality and quantity of food consumed by a donor influences its willingness to share with a hungry nestmate. For example, Farina and Nunez (1991) [27] used an assay with two nestmates similar to ours but that only measured trophallaxis behaviour and found that fully satiated bees were more willing to share food than partially fed or starved bees. In their assay, they showed that the sucrose concentration given to the donor had no effect on willingness to engage in trophallaxis of either donors or receivers.

Another study that examined the same social behaviours that we measured starved entire colonies of honeybees and observed the behaviour of the bees in the context of the hive. This study only found that begging frequency increased between nestmates in hungry colonies; other social behaviours including trophallaxis were unaffected [8]. In our experiments by contrast, hunger state had a strong influence on all behaviours associated with social interactions between two nestmates. As seen in ants [9], hungry honeybees were more likely to perform long bouts of begging. However, they were also less likely to give food via trophallaxis, to antennate, and to display mandible opening. The social behaviour of sucrose-fed bees, on the other hand, was characterized by antennation early in the observation period, food sharing, and a constant rate of mandible opening. These bees did not beg for food much in the beginning of the 30 min observation, but begged more later on, perhaps indicating that their hunger state was changing.

### Inebriation state and its influence on social behaviour

Mandible opening by honeybees during nestmate interactions has previously been interpreted as food offering behaviour that precedes trophallaxis with a nestmate [6], [8]. In contrast, studies of many insects including crickets [28], ants [29], [30], and bees [31], suggest that opening of the mandibles is a defensive posture that precedes a bite or sting attack when the animal recognizes an enemy [29], [31]. In sweat bees, mandible opening is also form of aggressive solicitation of food by dominant members of colonies [26], in which the dominant bee repeatedly opens and closes her mandibles and follows by nipping or biting. Non-aggressive food solicitation in this species is distinguished by the fact that it is preceded by antennation and then followed by extending the proboscis to beg for food [26] in a manner more similar to that described between honeybees [6].

In our study, mandible opening was not always followed by food offering behaviour. Hungry, sucrose-fed, and 5% ethanol-fed bees were equally likely to perform trophallaxis after opening their mandibles as they were to open their mandibles a second time. However, the bees fed 10% ethanol were 2 times more likely to perform bouts of mandible opening behaviour followed by a second bout of mandible opening than by trophallaxis. While we observed repeated mandible opening events, we did not observe nipping or biting between nestmates that might indicate that the inebriated bees were demanding food from the other bee in the arena. These bees were not motivated to seek food, as indicated by the fact that they were also the least likely to engage in food begging behaviour. This is corroborated by the observation in a previous study that bees fed 5% or 10% ethanol solutions were less motivated to eat [16].

The repeated mandible opening we observed may instead be a defensive posture on the part of the inebriated donor bee that perhaps indicates that it did not recognize the receiver bee as a nestmate or that it was unwilling to share food. Social insects antennate as a form of contact chemoreception which allows them to recognize nestmates and avoid or attack enemies [29], [32] and even facilitates food sharing [9]. We previously found that bees inebriated after feeding on 10% ethanol solutions had more difficulty distinguishing odours in an appetitive differential conditioning task in which they had to learn to differentiate odours associated with food from those that were not associated with a food reward [16]. Studies in *Drosophila* and *C. elegans* also suggest that ethanol affects olfactory processing in invertebrates [11], [33]. Here, inebriated donor bees were less likely to antennate other bees, and they might have also had more difficulty recognizing receiver's scent. For example, when ants do not recognize other ants as nestmates, they will not perform trophallaxis and instead open their mandibles as a form of warning display [29]. Although the inebriated bees were not on average less willing to engage in trophallaxis and transferred as much food as bees fed only sucrose, they did perform fewer bouts of trophallaxis and were less likely to beg for food from receivers. On average, the inebriated bees may have been less motivated to share food or to eat and thus were more likely to open their mandibles to reject begging from the receivers.

### Inebriation state and its influence on trophallaxis

Ethanol inebriation reduces motor coordination, affects sensory function, and influences the motivation to feed and the ability to learn in honeybees [15], [16], [34], most likely via interaction with several neurotransmitters and intracellular signalling processes [35]. Our analysis confirmed that measurement of an individual's motor function could predict whether the donor bees in our study had been fed ethanol. Being able to perform social behaviours also relies on motor coordination, so one might expect that ethanol inebriation would affect whether or not these behaviours were performed; indeed, the ethanol-fed bees performed less antennation than those fed sucrose. Other behaviours, such as trophallaxis and begging occurred less frequently than observed in sucrose-fed or hungry honeybees.

The inebriated bees in our study did not spend less time performing trophallaxis than the sucrose-fed bees, and transferred as much food to the receiver bees as the sucrose-fed bees. Therefore, there are at least two possible reasons why the behaviour of receiver bees was affected by donor bees having consumed ethanol. First, it is possible that the difference in the behaviour of the receivers arose from the reduced social activity of donors fed ethanol. For example, the receivers paired with donors given 5% ethanol may have shown reduced antennation due to the reduced level of antennation by donors in the 5% ethanol group. Alternatively, our data suggest that the receivers paired with ethanol-fed bees obtained food that contained ethanol via trophallaxis, as the receivers began to exhibit behaviours expected from inebriated bees, such as more grooming and less walking. This indicates not only that ethanol must have remained in the crop for more than 2 h after feeding, but that donors were also willing to share potentially dangerous food with nestmates. Taken together, these data indicate that donor bees are willing to share food containing ethanol with nestmates, even when they themselves have been poisoned or inebriated. It also indicates that hungry receivers will solicit food from nestmates that do not actively engage in the social behaviours that precede trophallaxis like antennation.

Our assay is the first to report in detail the behaviour exhibited by a donor bee during its social interactions with a hungry nestmate. Our assay is unique from earlier studies of the behaviour of nestmates in that we report the detailed behaviour of donors after carefully defining the physiological state of both the donor and receiver. We anticipate that this assay will aid researchers in studies designed to quantify how stress, disease, or drugs influence social interactions in social insects such as bees on the scale of two individuals.

## Supporting Information

Figure S1 **Individual behaviours of receivers towards 1.0 M sucrose-fed and 5% ethanol-fed donors.** (A, B) The receivers performed more bouts of walking later in the interval (Pois. Reg. χ_5_^2^ = 12.3, p = 0.031), but there was no effect of treatment (Pois. Reg χ_1_^2^ = 1.05, p = 0.307). The average bout duration for the stopped behaviour did not change during the interval for the receivers (interval main effect (GLM), F_1,19_ = 0.26, p = 0.614), and was unaffected by treatment (treatment main effect (GLM), F_1,19_ = 0.05, p = 0.823). However, the number times that the receivers stopped generally became more frequent later in the interval for the receiver with the sucrose bee (interaction, Pois. Reg. χ_5_ = 23.1, p<0.001). (C, D) Bouts of stopped behaviour depended on both interval and treatment (interaction, Pois. Reg. χ_5_ = 23.1, p<0.001).The mean duration of bouts of stopped behaviour did not change during the interval for the receivers (interval main effect, GLM, F_1,19_ = 0.26, p = 0.614), and was not affected by treatment (treatment main effect, GLM, F_1,19_ = 0.05, p = 0.823). (E, F) During the observation, the bout duration of which the receiver was upside down did not change (interval main effect, GLM, F_1,19_ = 0.96, p = 0.340), and was not significantly influenced by the treatments (main effect of treatment, GLM, F_1,19_ = 0.48, p = 0.498). The receiver bee with the ethanol-donor turned upside down more frequently later in the interval, but this trend was not observed for the receiver interacting with the sucrose-donor (interaction: Pois. Reg. χ_5_^2^ = 22.6, p<0.001, interval: Pois. Reg. χ_5_^2^ = 12.5, p = 0.029). Treatment did not affect how frequently the receivers went upside down (Pois. Reg. χ_1_^2^ = 0.961, p = 0.327). (G,H) The bouts of grooming in the receiver became shorter over the interval (interval main effect, GLM, F_1,19_ = 34.9, p<0.001), and were unaffected by dose (treatment main effect, GLM, F_1,19_ = 2.66, p = 0.119). Frequency of grooming bouts also decreased over time for the receiver (Pois. Reg. χ_5_^2^ = 15.1, p = 0.010) but were not affected by treatment (Pois. Reg. χ_5_^2^ = 0.56, p = 0.456).
(TIF)

Figure S2 **Social behaviour of receivers towards 1.0 M sucrose-fed and 5% ethanol-fed donors.** (A,B) The number of bouts of antennation remained constant during the interval (Pois. Reg χ_5_^2^ = 6.65, p = 0.248), and was not significantly affected by the treatment (Pois. Reg χ_1_^2^ = 3.31, p = 0.069). Mean bout duration of antennation changed over the interval for the receiver (interval main effect, GLM, F_1,19_ = 9.39, p = 0.006); the receiver with the sucrose-donor had longer bouts of antennation at the start of the interval, whereas the receiver with the ethanol-donor had bouts of the same duration over the observation period (interaction main effect, GLM, F_1,19_ = 9.36, p = 0.006). (C, D) The receivers begged (‘proboscis out’) more frequently later in the interval (Pois. Reg., χ_5_^2^ = 16.1, p = 0.006), but treatment did not affect the number of begging bouts of receivers (Pois. Reg., χ_1_^2^ = 0.35, p = 0.554).The average bout duration of begging behaviour was constant over time in receivers (interval main effect, GLM, F_1,19_ = 0.73, p = 0.405), bouts were of the same duration for both treatments (treatment main effect, GLM, F_1,19_ = 0.09, p = 0.772). (E, F) The bout duration of trophallaxis by the receivers were generally of the same length over the interval (interval main effect, GLM, F_1,19_ = 3.38, p = 0.082), and were also unaffected by treatment (treatment main effect, GLM, F_1,19_ = 1.36, p = 0.258). The frequency of trophallaxis bouts in donors remained the same over the interval (Pois. Reg χ_5_^2^ = 4.82, p = 0.438) once again this was not affected by dose (Pois. Reg χ_3_ = 0.40, p = 0.530). (G, H) For the receivers with the ethanol-donors, the bouts of mandible opening behaviour became more frequent later in the interval (Pois. Reg. χ_5_^2^ = 39.8, p<0.001), yet overall there was no effect of treatment on the number of bouts (Pois. Reg. χ_1_^2^ = 0.66, p = 0.416).Bouts of mandible opening behaviour were generally of equal length throughout the observation (interval main effect, GLM, F_1,19_ = 0.02, p = 0.889), and were the same for receivers with the sucrose-donor and the ethanol-donor (treatment main effect (GLM), F_1,19_ = 0.02, p = 0.900).
(TIF)

Table S1 **Factor Analysis for All Behavioural Variables.**
(DOCX)

Table S2 **Canonical Discriminant Analysis for Individual Motor Behaviours.**
(DOCX)

Table S3 **Canonical Discriminant Analysis for Social Behaviours.**
(DOCX)

Data S1 **Data for Donors and Receivers.**
(XLSX)
