# Supplementary figures and images for: Physiological State Influences the Social Interactions of Two Honeybee Nest Mates
Source: PLoS One. 2012 Mar 9;7(3):e32677. doi: 10.1371/journal.pone.0032677 (PMC3302875; doi:10.1371/journal.pone.0032677)

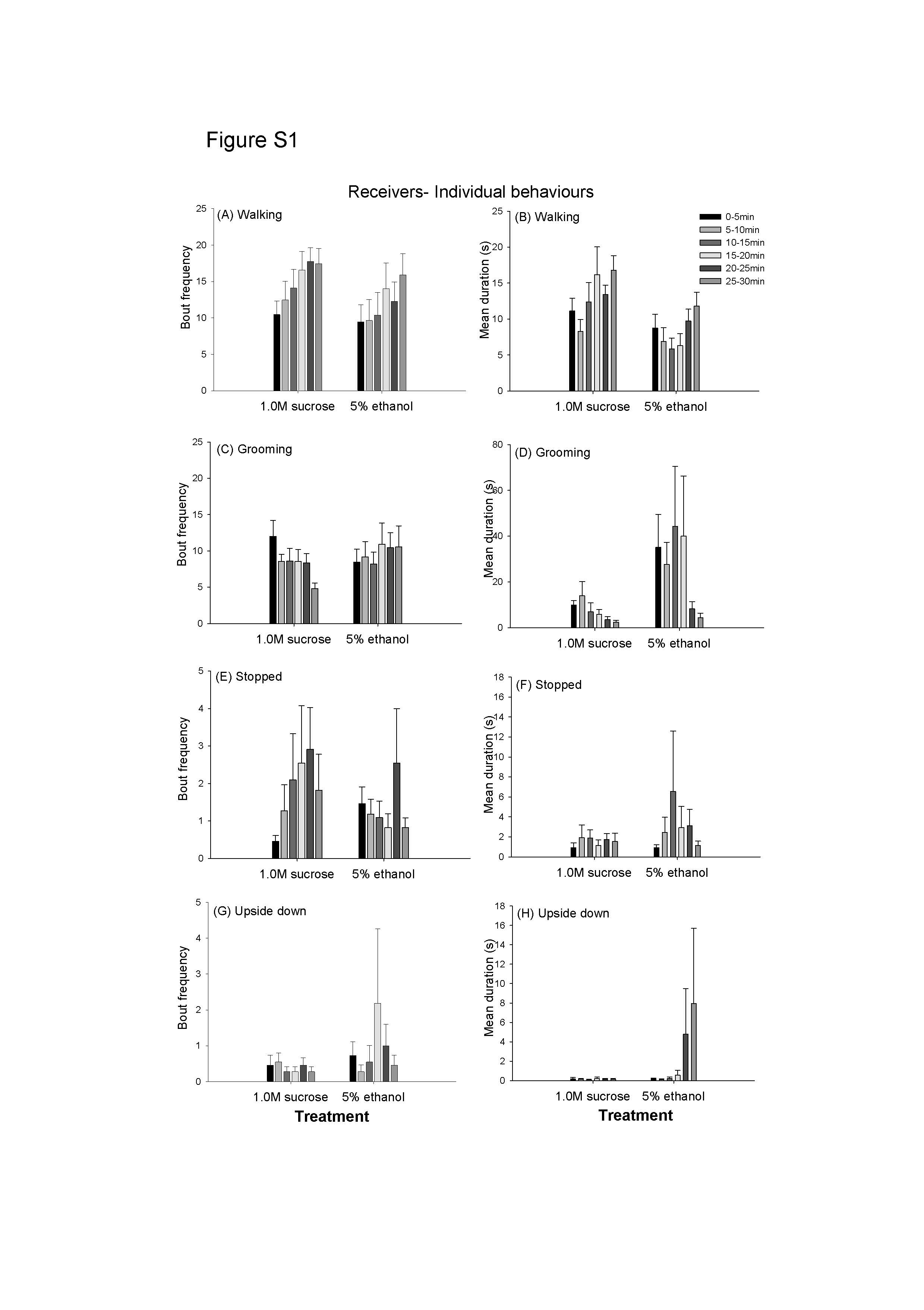

Supplement: Figure S1 — Individual behaviours of receivers towards 1.0 M sucrose-fed and 5% ethanol-fed donors. (A, B) The receivers performed more bouts of walking later in the interval (Pois. Reg. χ5 2 = 12.3, p = 0.031), but there was no effect of treatment (Pois. Reg χ1 2 = 1.05, p = 0.307). The average bout duration for the stopped behaviour did not change during the interval for the receivers (interval main effect (GLM), F1,19 = 0.26, p = 0.614), and was unaffected by treatment (treatment main effect (GLM), F1,19 = 0.05, p = 0.823). However, the number times that the receivers stopped generally became more frequent later in the interval for the receiver with the sucrose bee (interaction, Pois. Reg. χ5 = 23.1, p<0.001). (C, D) Bouts of stopped behaviour depended on both interval and treatment (interaction, Pois. Reg. χ5 = 23.1, p<0.001).The mean duration of bouts of stopped behaviour did not change during the interval for the receivers (interval main effect, GLM, F1,19 = 0.26, p = 0.614), and was not affected by treatment (treatment main effect, GLM, F1,19 = 0.05, p = 0.823). (E, F) During the observation, the bout duration of which the receiver was upside down did not change (interval main effect, GLM, F1,19 = 0.96, p = 0.340), and was not significantly influenced by the treatments (main effect of treatment, GLM, F1,19 = 0.48, p = 0.498). The receiver bee with the ethanol-donor turned upside down more frequently later in the interval, but this trend was not observed for the receiver interacting with the sucrose-donor (interaction: Pois. Reg. χ5 2 = 22.6, p<0.001, interval: Pois. Reg. χ5 2 = 12.5, p = 0.029). Treatment did not affect how frequently the receivers went upside down (Pois. Reg. χ1 2 = 0.961, p = 0.327). (G,H) The bouts of grooming in the receiver became shorter over the interval (interval main effect, GLM, F1,19 = 34.9, p<0.001), and were unaffected by dose (treatment main effect, GLM, F1,19 = 2.66, p = 0.119). Frequency of grooming bouts also decreased over time for the [file pone.0032677.s001.tif]

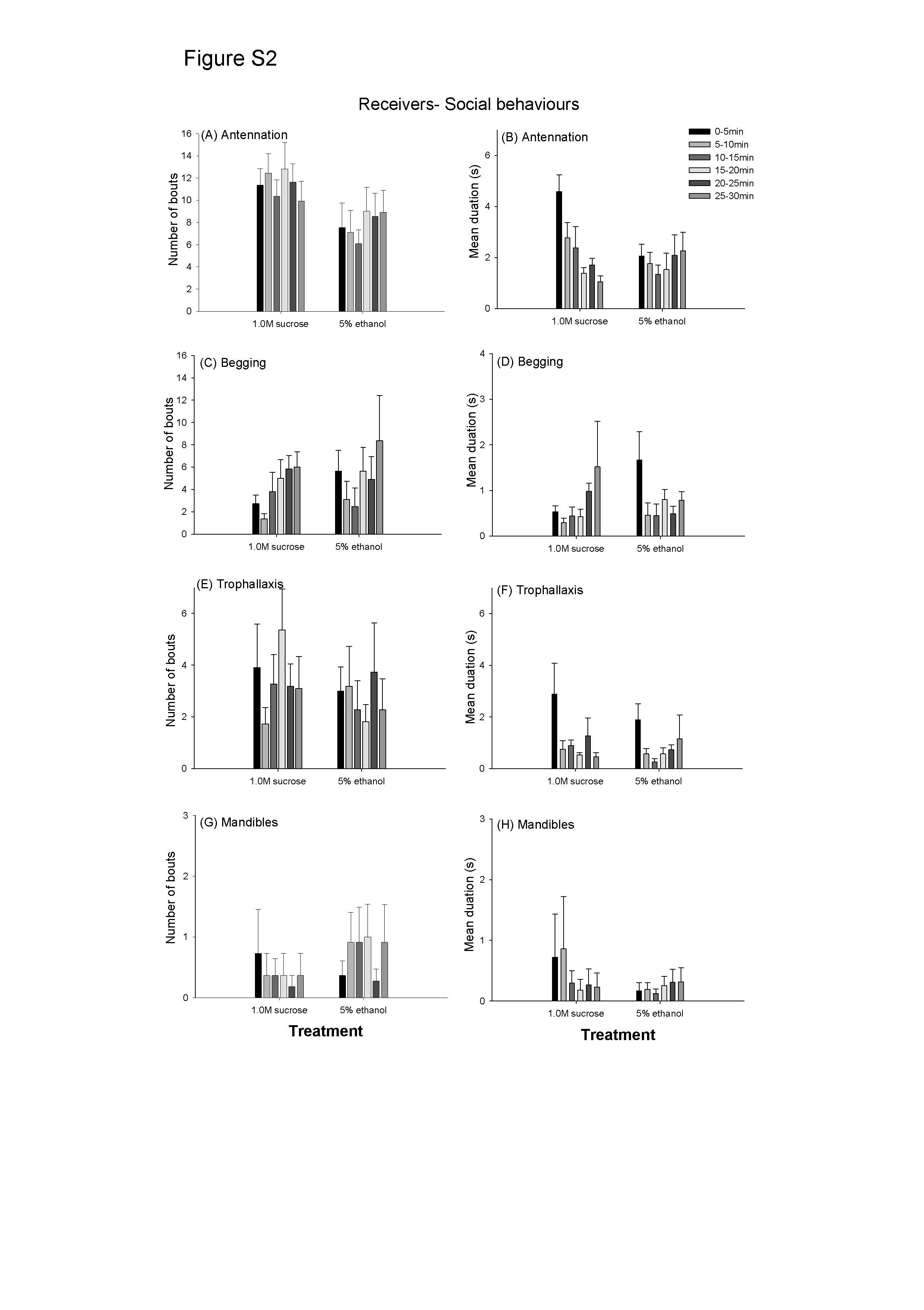

Supplement: Figure S2 — Social behaviour of receivers towards 1.0 M sucrose-fed and 5% ethanol-fed donors. (A,B) The number of bouts of antennation remained constant during the interval (Pois. Reg χ5 2 = 6.65, p = 0.248), and was not significantly affected by the treatment (Pois. Reg χ1 2 = 3.31, p = 0.069). Mean bout duration of antennation changed over the interval for the receiver (interval main effect, GLM, F1,19 = 9.39, p = 0.006); the receiver with the sucrose-donor had longer bouts of antennation at the start of the interval, whereas the receiver with the ethanol-donor had bouts of the same duration over the observation period (interaction main effect, GLM, F1,19 = 9.36, p = 0.006). (C, D) The receivers begged (‘proboscis out’) more frequently later in the interval (Pois. Reg., χ5 2 = 16.1, p = 0.006), but treatment did not affect the number of begging bouts of receivers (Pois. Reg., χ1 2 = 0.35, p = 0.554).The average bout duration of begging behaviour was constant over time in receivers (interval main effect, GLM, F1,19 = 0.73, p = 0.405), bouts were of the same duration for both treatments (treatment main effect, GLM, F1,19 = 0.09, p = 0.772). (E, F) The bout duration of trophallaxis by the receivers were generally of the same length over the interval (interval main effect, GLM, F1,19 = 3.38, p = 0.082), and were also unaffected by treatment (treatment main effect, GLM, F1,19 = 1.36, p = 0.258). The frequency of trophallaxis bouts in donors remained the same over the interval (Pois. Reg χ5 2 = 4.82, p = 0.438) once again this was not affected by dose (Pois. Reg χ3 = 0.40, p = 0.530). (G, H) For the receivers with the ethanol-donors, the bouts of mandible opening behaviour became more frequent later in the interval (Pois. Reg. χ5 2 = 39.8, p<0.001), yet overall there was no effect of treatment on the number of bouts (Pois. Reg. χ1 2 = 0.66, p = 0.416).Bouts of mandible opening behaviour were generally of equal length throughout the observation (interval main effect, GLM, F1,19 = 0 [file pone.0032677.s002.tif]
