# Supplementary material for: Physiological State Influences the Social Interactions of Two Honeybee Nest Mates
Source: PLoS One. 2012 Mar 9;7(3):e32677. doi: 10.1371/journal.pone.0032677 (PMC3302875; doi:10.1371/journal.pone.0032677)
Supplement: Table S1 — Factor Analysis for All Behavioural Variables. (DOCX) [file pone.0032677.s003.docx]

**Table S1**

Factor Analysis for All Behavioural Variables (% of time spent for each 5 min interval)

| Factors | | | | | | | | | | | | | |
| --- | --- | --- | --- | --- | --- | --- | --- | --- | --- | --- | --- | --- | --- |
|  | 1 | 2 | 3 | 4 | 5 | 6 | 7 | 8 | 9 | 10 | 11 | 12 | 13 |
| Eigenvalues | 9.621 | 5.260 | 4.714 | 4.441 | 2.990 | 2.225 | 1.972 | 1.613 | 1.529 | 1.374 | 1.255 | 1.189 | 1.094 |
| % variance | 20.044 | 10.958 | 9.822 | 9.251 | 6.230 | 4.635 | 4.108 | 3.361 | 3.186 | 2.863 | 2.614 | 2.477 | 2.278 |
|  |  |  |  |  |  |  |  |  |  |  |  |  |  |
| **Factor loading** |  |  |  |  |  |  |  |  |  |  |  |  |  |
| W1 | **-0.480** | -0.088 | 0.217 | -0.061 | -0.054 | 0.222 | -0.018 | 0.562 | -0.052 | -0.296 | 0.225 | -0.184 | 0.259 |
| W2 | **-0.812** | 0.091 | 0.052 | -0.213 | -0.038 | -0.138 | -0.104 | 0.073 | -0.113 | -0.239 | -0.048 | 0.063 | -0.016 |
| W3 | **-0.834** | 0.040 | 0.150 | -0.081 | -0.024 | -0.102 | 0.029 | -0.165 | -0.050 | 0.043 | 0.172 | 0.110 | 0.111 |
| W4 | **-0.868** | -0.015 | 0.168 | -0.011 | -0.030 | -0.136 | 0.047 | -0.111 | -0.049 | 0.067 | 0.024 | 0.185 | 0.107 |
| W5 | **-0.814** | -0.062 | 0.227 | -0.054 | -0.211 | -0.007 | 0.176 | 0.021 | 0.104 | 0.192 | -0.018 | -0.043 | 0.154 |
| W6 | **-0.711** | -0.076 | 0.313 | -0.043 | -0.172 | 0.021 | 0.227 | 0.141 | 0.003 | 0.341 | -0.192 | 0.040 | -0.044 |
| S1 | 0.376 | -0.062 | -0.018 | 0.073 | 0.180 | -0.395 | 0.560 | 0.147 | 0.153 | -0.216 | 0.119 | 0.289 | 0.053 |
| S2 | 0.508 | -0.157 | -0.044 | -0.126 | 0.298 | -0.228 | 0.567 | 0.130 | -0.003 | -0.057 | -0.005 | -0.221 | -0.158 |
| S3 | 0.310 | -0.097 | -0.195 | -0.066 | -0.023 | -0.006 | 0.560 | 0.139 | -0.246 | 0.288 | -0.155 | -0.371 | -0.070 |
| S4 | 0.456 | -0.065 | -0.267 | -0.114 | -0.067 | 0.207 | 0.013 | 0.142 | -0.468 | 0.008 | -0.216 | -0.109 | 0.253 |
| S5 | 0.572 | -0.046 | -0.081 | 0.044 | 0.001 | 0.070 | 0.027 | -0.096 | -0.424 | -0.010 | -0.139 | 0.332 | 0.140 |
| S6 | 0.317 | 0.051 | -0.337 | -0.022 | 0.272 | -0.092 | 0.501 | -0.033 | -0.038 | -0.012 | 0.113 | 0.115 | 0.366 |
| U1 | -0.225 | **-0.640** | -0.286 | **0.545** | 0.111 | 0.073 | -0.081 | -0.008 | 0.042 | -0.040 | -0.116 | -0.131 | 0.001 |
| U2 | -0.224 | **-0.628** | -0.299 | **0.574** | 0.113 | 0.066 | -0.067 | -0.018 | 0.071 | -0.029 | -0.105 | -0.098 | 0.009 |
| U3 | -0.331 | **-0.522** | -0.364 | **0.633** | 0.152 | 0.009 | -0.009 | -0.059 | 0.044 | -0.065 | -0.074 | -0.002 | -0.004 |
| U4 | -0.354 | **-0.668** | -0.351 | **0.441** | 0.080 | -0.003 | -0.031 | -0.079 | 0.039 | -0.023 | -0.032 | 0.027 | -0.022 |
| U5 | -0.307 | **-0.596** | -0.372 | **0.502** | 0.053 | 0.097 | 0.068 | 0.026 | 0.025 | 0.041 | 0.002 | 0.015 | -0.055 |
| U6 | -0.314 | **-0.467** | -0.297 | **0.378** | -0.042 | 0.004 | 0.190 | -0.013 | 0.035 | 0.108 | 0.194 | 0.183 | 0.064 |
| G1 | **0.630** | -0.013 | -0.043 | -0.023 | -0.286 | -0.169 | 0.055 | -0.481 | 0.106 | 0.232 | -0.110 | 0.021 | -0.076 |
| G2 | **0.838** | 0.008 | -0.036 | 0.157 | -0.214 | 0.148 | 0.019 | -0.038 | 0.047 | 0.255 | 0.175 | -0.058 | 0.071 |
| G3 | **0.840** | -0.087 | -0.144 | 0.065 | -0.072 | 0.085 | -0.260 | 0.087 | 0.069 | -0.014 | -0.104 | 0.045 | 0.065 |
| G4 | **0.860** | -0.088 | 0.008 | 0.105 | -0.088 | -0.006 | 0.000 | 0.100 | 0.230 | 0.031 | -0.040 | 0.042 | -0.157 |
| G5 | **0.852** | -0.083 | -0.172 | 0.040 | -0.026 | -0.060 | -0.165 | 0.056 | 0.064 | -0.074 | 0.028 | 0.055 | 0.161 |
| G6 | **0.709** | -0.039 | -0.302 | -0.042 | 0.081 | -0.103 | -0.353 | -0.064 | 0.167 | -0.209 | 0.129 | 0.015 | 0.138 |
| A1 | -0.143 | 0.399 | 0.346 | 0.376 | 0.266 | 0.122 | 0.026 | -0.227 | -0.208 | -0.018 | -0.186 | 0.090 | 0.206 |
| A2 | -0.097 | 0.456 | 0.271 | 0.342 | 0.013 | 0.321 | 0.093 | -0.166 | 0.245 | -0.098 | -0.305 | 0.032 | 0.275 |
| A3 | 0.146 | 0.201 | 0.280 | 0.273 | 0.195 | 0.271 | 0.263 | 0.081 | 0.468 | -0.203 | 0.120 | 0.199 | 0.011 |
| A4 | -0.061 | 0.247 | 0.367 | 0.192 | -0.036 | 0.304 | 0.095 | -0.351 | 0.408 | -0.007 | -0.004 | -0.356 | 0.113 |
| A5 | 0.243 | 0.190 | 0.319 | 0.339 | -0.126 | 0.537 | 0.228 | 0.243 | 0.106 | 0.009 | 0.070 | -0.002 | -0.191 |
| A6 | 0.287 | 0.195 | 0.245 | 0.462 | -0.032 | 0.552 | 0.017 | 0.165 | -0.255 | 0.096 | -0.065 | 0.180 | -0.138 |
| P1 | -0.146 | 0.255 | **-0.497** | -0.261 | 0.549 | 0.244 | -0.083 | -0.022 | -0.013 | 0.048 | 0.090 | -0.077 | 0.095 |
| P2 | -0.154 | 0.266 | **-0.475** | -0.286 | 0.563 | 0.243 | -0.036 | 0.073 | 0.152 | 0.247 | -0.018 | 0.043 | 0.148 |
| P3 | -0.332 | 0.326 | **-0.393** | -0.163 | 0.279 | -0.007 | -0.033 | 0.015 | 0.138 | 0.352 | -0.044 | 0.211 | -0.351 |
| P4 | -0.153 | 0.345 | **-0.532** | -0.153 | 0.554 | 0.239 | 0.025 | -0.080 | 0.024 | 0.208 | 0.109 | -0.022 | 0.149 |
| P5 | -0.180 | 0.411 | **-0.425** | -0.031 | 0.355 | 0.002 | -0.128 | 0.042 | 0.049 | -0.273 | -0.051 | -0.060 | -0.365 |
| P6 | -0.209 | 0.625 | **-0.123** | 0.367 | 0.230 | 0.019 | -0.006 | -0.005 | -0.235 | -0.208 | -0.055 | -0.192 | -0.222 |
| T1 | -0.044 | 0.377 | -0.029 | **0.522** | 0.096 | -0.356 | -0.237 | 0.201 | 0.016 | 0.336 | -0.016 | -0.021 | 0.175 |
| T2 | 0.130 | 0.315 | 0.037 | **0.292** | -0.041 | -0.447 | -0.212 | 0.264 | 0.296 | 0.276 | -0.210 | -0.090 | 0.138 |
| T3 | -0.008 | 0.517 | 0.075 | **0.586** | 0.087 | -0.301 | -0.013 | 0.176 | -0.061 | -0.062 | -0.198 | 0.086 | 0.009 |
| T4 | 0.033 | 0.404 | 0.035 | **0.491** | -0.102 | -0.227 | 0.007 | -0.167 | -0.191 | 0.015 | 0.552 | -0.073 | 0.022 |
| T5 | 0.058 | 0.541 | 0.040 | **0.595** | 0.121 | -0.346 | 0.106 | 0.100 | -0.088 | -0.114 | -0.162 | 0.025 | -0.029 |
| T6 | 0.092 | 0.274 | 0.025 | **0.543** | -0.030 | 0.039 | 0.005 | -0.350 | -0.245 | 0.090 | 0.389 | -0.208 | -0.083 |
| M1 | 0.188 | -0.117 | **0.394** | 0.171 | 0.163 | 0.130 | -0.189 | 0.365 | -0.033 | 0.331 | 0.321 | 0.253 | -0.108 |
| M2 | 0.151 | -0.284 | **0.551** | -0.104 | 0.534 | -0.229 | 0.046 | -0.107 | -0.023 | 0.010 | 0.020 | 0.017 | -0.054 |
| M3 | 0.210 | -0.453 | **0.547** | 0.073 | 0.474 | 0.009 | -0.191 | -0.012 | -0.149 | 0.036 | 0.010 | 0.110 | -0.017 |
| M4 | 0.147 | -0.307 | **0.519** | -0.010 | 0.367 | -0.105 | -0.213 | 0.190 | -0.016 | 0.110 | 0.052 | -0.368 | 0.107 |
| M5 | 0.146 | -0.274 | **0.621** | -0.068 | 0.571 | -0.175 | -0.019 | -0.091 | 0.025 | 0.019 | -0.008 | -0.155 | -0.024 |
| M6 | 0.233 | -0.310 | **0.578** | -0.061 | 0.391 | 0.132 | 0.001 | -0.274 | -0.108 | -0.020 | -0.160 | 0.146 | -0.058 |

W = walking, S = stopped, G = grooming, U = upside down, A = antennate, P = proboscis out (begging), T = trophallaxis, M = mandibles open. Each number indicates the interval (1=0-5min, 2=5-10min, 3=10-15min, 4=15-20min, 5=20-25min, 6=25-30min). The top portion of the table indicates the eigenvalues and % variance contributed by each factor. The bottom half of the table indicates the loading for each variable on each factor. Factor loadings in bold indicate the behaviours with the greatest contribution to each factor for the first 4 factors.
