# Supplementary material for: Physiological State Influences the Social Interactions of Two Honeybee Nest Mates
Source: PLoS One. 2012 Mar 9;7(3):e32677. doi: 10.1371/journal.pone.0032677 (PMC3302875; doi:10.1371/journal.pone.0032677)
Supplement: Table S3 — Canonical Discriminant Analysis for Social Behaviours. (DOCX) [file pone.0032677.s005.docx]

Supplementary Table 3

**Canonical Discriminant Analysis for Social Behaviours**

|  | Discriminant function | | |
| --- | --- | --- | --- |
|  | 1 | 2 | 3 |
| Eigenvalue | 0.567 | 0.204 | 0.080 |
| % variance | 66.600 | 24.000 | 9.400 |
| Wilks' Lambda | 0.491 | 0.769 | 0.926 |
|  |  |  |  |
| **Factors** |  |  |  |
| F1 | -0.010 | **0.621** | -0.009 |
| F2 | **0.970** | -0.058 | -0.167 |
| F3 | 0.270 | 0.251 | 0.309 |
| F4 | -0.105 | -0.392 | -0.342 |
| F5 | -0.049 | -0.423 | **0.666** |
| F6 | 0.138 | 0.427 | 0.476 |
| F7 | 0.242 | -0.418 | 0.322 |
|  |  |  |  |
| **Treatment** |  |  |  |
| No food | -1.005 | 0.247 | -0.101 |
| 1.0M sucrose | 0.936 | 0.429 | -0.160 |
| 5% Ethanol | 0.127 | -0.107 | 0.462 |
| 10% Ethanol | 0.157 | -0.801 | -0.241 |

The standardized coefficients in bold indicate the factors from the factor analysis that contributed the most to classification of the treatments by each of the functions (all factors contributed, but the criterion for emphasis was a coefficient greater than +0.5). The sign and magnitude of the non-standardized coefficients indicate how the functions classified bees according to treatment. The order of the functions indicates the distance in similarity between the treatments; the line indicates the most likely split between groups made by the classification function. Numbers in grey indicate neutral groups midway between the groups split by the secondary functions.
